# Complete Biodegradation of Diclofenac by New Bacterial Strains: Postulated Pathways and Degrading Enzymes

**DOI:** 10.3390/microorganisms11061445

**Published:** 2023-05-30

**Authors:** Mahmoud S. M. Mohamed, Ayan A. Asair, Nashwa A. H. Fetyan, Sherif M. Elnagdy

**Affiliations:** 1 Department of Botany and Microbiology, Faculty of Science, Cairo University, Giza 12613, Egypt; 2 Department of Microbiology, Soil, Water and Environment Research Institute, Agriculture Research Center, Giza 12619, Egypt

**Keywords:** *Achromobacter*, biodegradation, diclofenac, metabolite, *Pseudomonas*

## Abstract

The accumulation of xenobiotic compounds in different environments interrupts the natural ecosystem and induces high toxicity in non-target organisms. Diclofenac is one of the commonly used pharmaceutical drugs that persist in the environment due to its low natural degradation rate and high toxicity. Therefore, this study aimed to isolate potential diclofenac-degrading bacteria, detect the intermediate metabolites formed, and determine the enzyme involved in the degradation process. Four bacterial isolates were selected based on their ability to utilize a high concentration of diclofenac (40 mg/L) as the sole carbon source. The growth conditions for diclofenac degradation were optimized, and bacteria were identified as *Pseudomonas aeruginosa* (S1), *Alcaligenes aquatilis* (S2), *Achromobacter spanius* (S11), and *Achromobacter piechaudii* (S18). The highest percentage of degradation was recorded (97.79 ± 0.84) after six days of incubation for *A. spanius* S11, as analyzed by HPLC. To detect and identify biodegradation metabolites, the GC-MS technique was conducted for the most efficient bacterial strains. In all tested isolates, the initial hydroxylation of diclofenac was detected. The cleavage step of the NH bridge between the aromatic rings and the subsequent cleavage of the ring adjacent to or in between the two hydroxyl groups of polyhydroxylated derivatives might be a key step that enables the complete biodegradation of diclofenac by *A. piechaudii* S18, as well as *P. aeruginosa* S1. Additionally, the laccase, peroxidase, and dioxygenase enzyme activities of the two *Achromobacter* strains, as well as *P. aeruginosa* S1, were tested in the presence and absence of diclofenac. The obtained results from this work are expected to be a useful reference for the development of effective detoxification bioprocesses utilizing bacterial cells as biocatalysts. The complete removal of pharmaceuticals from polluted water will stimulate water reuse, meeting the growing worldwide demand for clean and safe freshwater.

## 1. Introduction

The continuous growth of the world population is increasing the demand for water supply throughout the world. The number of areas on the Earth that are suffering from a water crisis is growing for many reasons, such as climatic change, war, and the contamination of groundwater. Recently, several micropollutants were detected in water, such as pharmaceuticals, industrial chemicals, personal care products, and many other xenobiotic compounds. These pollutants have become a significant problem in the aquatic environment around the world [1]. In fact, the immoderate usage of pharmaceuticals in human and veterinary medicine is expanding its occurrence in the environment. In this regard, many types of pharmaceutical compounds have been found in surface water, groundwater, and even in drinking water [2]. 

Diclofenac (DCF), a pharmaceutical compound, is one of the most frequently used non-steroidal anti-inflammatory drugs. It is used to treat pain, fever, and inflammation and can be used without a prescription. The fate of DCF after human metabolism and excretion in the urine and feces is as metabolites, along with the unaltered parent compounds, which may be subjected to further transformations in wastewater treatment plants (WWTPs), producing metabolites that may be more toxic than DCF [3]. In several monitoring studies, DCF was detected in wastewater in several countries [4], and the occurrence of DCF in drinking water was also reported [5,6]. The presence of DCF in the aquatic environment is anticipated to be a long-term concern due to its potential toxic effects on non-target organisms [7,8]. In the aquatic environment, a high occurrence percentage of DCF was reported, and the detected concentrations of DCF were found to be in a range from 24 to 1043 ng/L in surface water [9,10]. DCF was found at a high concentration in the effluent water of sewage treatment plants (i.e., treated effluent) because the rate of DCF elimination in WWTPs is low and does not exceed 40%, despite the progress in technologies for wastewater treatment [11]. Therefore, the European Environmental Agency and the scientific community consider DCF to be a particular environmental concern (Directive 2013/39/EU).

The degradation of DCF by microorganisms is a cost-efficient, eco-friendly alternative solution. Only a few studies have reported the ability of bacteria to biodegrade DCF due to the deleterious effect of DCF on bacterial physiology, such as membrane injury, lipid peroxidation, and oxidative stress [7,12]. Furthermore, Facey et al. (2018) documented the aerobic detoxification of DCF by a microbial consortium from forest soil after 10 days in a low-salt medium into 2,6-dichloroaniline and carboxylated 2-hydroxyphenylacetic acid. Moreover, the microbial community in activated sludge showed the biodegradation of up to 21% of DCF in WWTPs through co-metabolic mechanisms and demonstrated the dominance of some bacterial genera, such as *Asticcacaulis*, *Pseudacidovorax*, and *Nitratireductor* after DCF exposure [13]. However, the first report on the degradation of DCF by a single bacterial strain revealed the biotransformation of DCF (1.7 μM) by *Labrys portucalensis* (F11) after 6 days, with the formation of benzoquinone imine as an intermediate metabolite in the degradation pathway, but complete degradation was only achieved by co-metabolism with acetate [14].

However, the specific bacterial mechanism responsible for degradation, the intermediate metabolites, and the enzymes involved in DCF degradation were unknown in most cases, and there are still gaps in the knowledge associated with the degradation process. Therefore, in the present study, different samples contaminated with DCF were used to explore the microbial diversity, isolating a potential local bacterial isolate that can effectively completely biodegrade high concentrations of DCF (40 mg/L) to less toxic or nontoxic compounds under optimized conditions. Moreover, the GC-MS technique was used to postulate the catabolic degradation pathways for DCF based on the detection of intermediate and residual compounds. In addition, the specific activities of enzymes involved in biodegradation were estimated. To our knowledge, this may be the first study to postulate a complete pathway for the biodegradation of high concentrations of DCF by three different bacterial isolates, with DCF being the sole carbon source.

## 2. Materials and Methods

### 2.1. Sample Collection and Isolation of Local Diclofenac-Degrading Bacteria

Soil samples were collected from contaminated Nile River water and soil from Gazira Park (Giza, Egypt). Other samples were collected from contaminated soil from the upper (20–30 cm) layer of a vegetable field located at Kaliobia Governorate, Egypt. The enrichment of DCF-degrading bacteria was performed by inoculating each sample (10 g of soil (wet weight) or 10 mL of water) into 100 mL of the minimal salt medium (MS) containing the following (g/L): K_2_HPO_4_ (2.0), KH_2_PO_4_ (1.0), MgSO_4_·7H_2_O (0.25), NH_4_So_4_ (0.5), and trace elements, including FeSO_4_·7H_2_O (0.05), MnSO_4_·4H_2_O (0.05), and CuSO_4_·5H_2_O (0.05), supplemented with 200 mg/L DCF [15]. The flasks were incubated at 37 °C for 24 h in a static incubator. In the enrichment culture, all strains used DCF as the sole source of carbon and energy. One milliliter of enriched bacterial culture was serially diluted up to a 10^−6^ dilution. Then, 100 µL was plated onto solid MS medium with DCF (200 µg/mL) and incubated at 37 °C for 48 h. Morphologically different bacterial isolates were selected, purified, and maintained at 4 °C on nutrient agar slants and glycerol stocks at −20 °C for further investigation. These isolates were named diclofenac-degrading strains (S1–S23) and used in the following experiments.

### 2.2. Optimization of Bacterial Isolate Growth under Different Physicochemical Conditions

Each bacterial isolate was tested for its ability to grow at different concentrations of DCF (10, 20, 30, and 40 mg/L) as the sole carbon source, and isolates were enumerated by viable counts using Luria Bertani agar medium (Laboratories Conda SA, Madrid, Spain) at pH 7.0 after aerobic incubation at 37 °C for 24 h. Bacterial growth was confirmed by Gram staining; the experiment was repeated three times in the same conditions, and the viable count was performed in all samples in triplicate. The counted bacterial colonies were expressed as log_10_ CFU/mL and plotted against the time of incubation [16].

Based on the preliminary experiment, the initial conditions of the biodegradation experiment were set as follows: MS medium was supplemented with a DCF concentration of 10 mg/L, the pH was adjusted to 7, and incubation was carried out with 150 rpm shaking at 28 °C. The concentration used was 100–400 times higher than the maximum recorded concentration detected in urban wastewater, i.e., 95 μg/L [1].

MS medium was inoculated with 5 mL of an overnight culture of each bacterium and then divided into four flasks, and each flask was supplemented with one of the selected concentrations. Bacterial growth was measured at 600 nm using a spectrophotometer (Jenway 6305; Bibby Scientific Ltd., Staffordshire, UK) after 1–7 days of incubation. The experiment was repeated three times, and the average reading was calculated. Similarly, DCF biodegradation for each bacterial isolate was monitored at different temperatures (28, 37, and 40 °C) and different pH values (4, 5, 6, 7, 8, and 9) after 1–7 days of incubation. Oxygen is sometimes essential for cell growth and biodegradation. Finally, to optimize the oxygen demand of each bacterial isolate, the cultures were incubated under static and shaking conditions (150 rpm) in MS medium supplemented with 10 mg/L DCF, and the cell density was evaluated.

### 2.3. Biochemical Characterization and Molecular Identification of the Selected Bacterial Isolates

A single colony from each bacterial isolate was examined for colony shape, size, and pigmentation. Gram reactions of all isolates were recorded. All bacterial isolates were categorized for the different physiological and biochemical tests. The isolates were first identified to the genus level according to biochemical tests described previously in detail [17], and then 16S rDNA sequence analysis was utilized for identification, as previously described [18]. The sequence reads obtained from Macrogen (Seoul, Republic of Korea) were assembled and trimmed, and the contig sequence for each isolate was blasted against the reference 16S rRNA gene sequences of other bacteria deposited in GenBank. The phylogenetic tree was generated using the maximum likelihood method via the MEGAX program with 1000 bootstrap replications [19].

### 2.4. Quantification of Diclofenac Biodegradation

The percentage of DCF degradation was calculated after measuring the residual DCF concentration for each bacterial isolate using high-performance liquid chromatography (HPLC) according to a validated method previously described by Nguyen et al. [13]. In summary, MS broth medium supplemented with 10 mg/L DCF (500 mL) was used as a basic medium. The medium was divided into five flasks; each flask was inoculated with a bacterial isolate (5% overnight grown culture), and one flask was used as a negative control (without bacteria). Samples were collected after three and six days of incubation and centrifuged at 8000× *g* for 10 min, and the cell-free supernatant was filtered using a 0.45 µm membrane filter (Millipore Corp., Burlington, MA, USA). DCF was measured at a wavelength of 270 nm. The sample volume injected into the HPLC instrument was 100 μL, and the detection limit was 10 μg/L. The percentage of DCF degradation was calculated using the following equation:

$$\mathrm{Percentage}\;\mathrm{of}\;\mathrm{degradation}\;=\left(\mathrm{Ci}-\mathrm{Cf}\right)\times \frac{100}{\mathrm{Cf}}$$

where Ci is the concentration of DCF at the beginning of the experiment, and Cf is the concentration at the end of the experiment (the cell-free supernatant of the sample).

### 2.5. Detection of Diclofenac Metabolites Produced by Individual Bacterial Isolates Using Gas Chromatography–Mass Spectrometry (GC–MS) Analysis

The selected bacterial isolate extracts were analyzed using GC–MS to identify the metabolic products of DCF during degradation in MS according to the method previously described by Ivshina et al. [20]. Chromatographic analyses were performed using a Finnigan Mat GCQ GC/ITD-MS. Helium was used as a carrier gas with the flow rate adjusted to constant velocity (40 cm/S). The injector temperature was 250 °C, and 2 μL of the sample was injected in the splitless injection mode adjusted to a splitless time of 0.8 min on. The oven temperature was programmed to 100 °C for 1 min following injection, after which the temperature was increased (30 °C/min) to 150°C, maintained for 1 min, then increased to 205 °C (3 °C/min), and finally increased to 260 °C (10 °C/min) for 23 min. The electron energy for the mass spectra was set at 70 V, and 200 °C was the ion source temperature. The ITD settings were as follows: mass range of 50–500, 3 microscans, and max ion time of 25 ms. The metabolites were detected and identified by comparing their retention times and mass spectra with the mass spectral data detailed in the library of WILEY, National Institute of Standard and Technology (NIST-11), and the mass spectral data of DCF metabolites in the literature.

### 2.6. Enzyme Activities

The bacterial isolates were first grown in 50 mL of MS broth supplemented with 10 mg/L DCF or 10 mg/L glucose as a control for 48 h. Laccase, catechol 1,2-dioxygenase, catechol 2,3-dioxygenase, and peroxidase activities were assayed using cell-free culture supernatants (extracellular activities). The reaction mixture for the determination of laccase activity consisted of 2 mL of 10% ABTS in 100 mM acetate buffer (pH 4.9), and the increase in optical density at 420 nm was recorded [21]. Catechol 1,2-dioxygenase and catechol 2,3-dioxygenase were assayed spectrophotometrically based on the formation of cis, cis-muconic acid at 260 nm (ε260 = 16,800/M cm) and the formation of 2-hydroxymuconic semialdehyde at 375 nm (ε375 = 36,000/M cm), respectively [22].

Peroxidase activities were measured using a procedure that determines the rate of pyrogallol decomposition [23]. All enzyme assays were conducted at 37 (or 30) °C, where the control was tested in the presence of glucose in the medium instead of DCF, and the reference blank contained all components but with the boiled enzyme. All enzyme assays were carried out three times and the average reading was calculated. The unit of enzyme was defined as a change in absorbance unit/min/mg of protein.

### 2.7. Statistical Analysis

The data obtained are presented in figures and tables as the mean value ± standard deviation (SD) of at least three replicates. A one-way ANOVA setting followed by Tukey’s HSD test (Minitab 18) was used to identify the differences between different treatments, and differences are represented by letters. Different letters are considered statistically significant differences at *p* ≤ 0.05.

## 3. Results

### 3.1. Screening for Local Diclofenac-Degrading Bacteria

To isolate bacteria capable of degrading diclofenac, four different polluted soil and water samples were used. Twenty-three bacterial isolates (S1–S23) were selected and purified based on their ability to utilize diclofenac as a sole carbon source. In order to select the most active isolates, the ability to degrade diclofenac at a higher concentration (10-fold) was further evaluated in minimal salt medium supplemented with 2000 µg/mL diclofenac. The results revealed that only four morphologically different bacterial isolates (S1, S2, S11, and S18) were able to grow after 24 h and therefore were selected and further characterized.

### 3.2. Ideal Growth Parameters for Diclofenac-Degrading Bacteria

**Figure 1:**
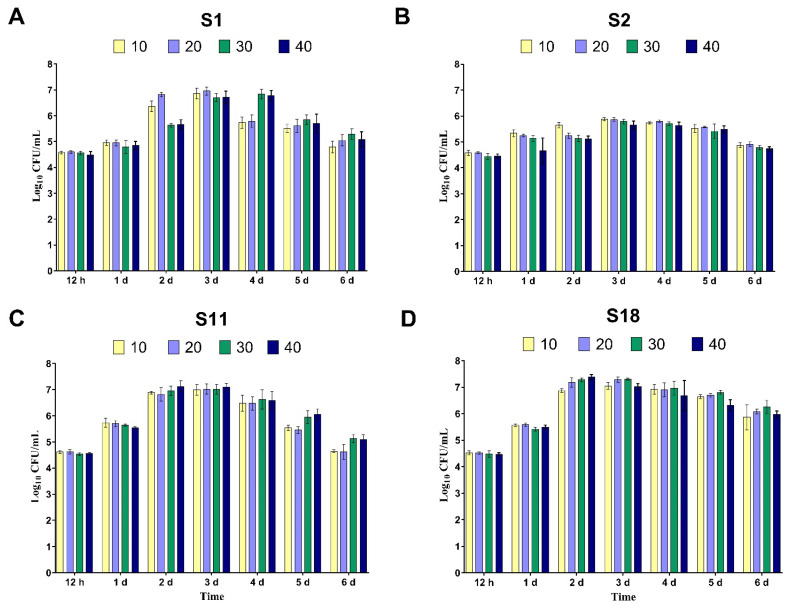
The growth of the four selected bacterial isolates ((**A**), S1; (**B**), S2; (**C**), S11; and (**D**), S18) determined by the viable count method (log_10_ CFU/mL) after different time intervals (1–7 days) of bacterial growth in minimal salt media supplemented with different concentrations of diclofenac (10, 20, 30, and 40 mg/L) as a single carbon source. The bars represent mean ± SD of three independent replicates.

Each isolate was tested individually for optimal growth conditions with diclofenac as the sole carbon source. The results revealed that all bacterial isolates could grow efficiently at all tested concentrations and times. The rise in DCF concentration delayed the maximum growth reached by all bacteria. The maximum growth was recorded after three days of incubation for all isolates (Figure 1A–D). At higher concentrations of DCF, i.e., 30 and 40 mg/L, isolates S1 and S2 showed a delayed lag phase, while S11 and S18 underwent exponential growth after only two days of incubation, and the viable counts started to decline after 5 days (Figure 1C,D). Isolate S2 showed relatively moderate growth during all incubation times.

In order to decrease the effect of different environmental growth conditions on DCF biodegradation, deviations from the best bacterial growth conditions, including temperature, pH, and shaking incubation, were evaluated. The effect of temperature on DCF degradation demonstrated that 37 °C is the best temperature for the growth of all isolates relative to other tested growth temperatures, except S1, which had the maximum growth at 28 °C after 4 days (Supplementary File, Figure S1). The selected isolates were further tested for biodegradation conditions at different pH values, ranging from 5 to 9. The results revealed that a neutral or slightly alkaline pH is the optimal pH for the growth and biodegradation of DCF for all isolates except isolate S2, which grew better at acidic pH (pH 5 and 6) (Figure S2).

On the other hand, the effect of static and shaking incubation conditions on the performance of bacterial culture was monitored after different time intervals (1–6 days). The results showed that bacterial growth was higher under shaking conditions (inferior under static conditions) for isolates S11 and S18 (Figure S3c,d). The growth of isolates S1 and S2 increased after 2 days of static incubation, with maximum growth reached at that time, and then the growth gradually decreased (Figure S3a,b), but with shaking incubation, the growth began to increase more than under static conditions after 3 days and reached the maximum for isolate S1 after 5 days and S2 after 3 days.

### 3.3. Removal Efficiency of Isolated Bacterial Strains

The optimal conditions obtained in previous experiments were used to determine the efficiency of DCF degradation. The amount of residual DCF remaining in each of the selected bacterial cultures was determined by HPLC analysis (Figure S4). DCF concentrations were considerably reduced only after 3 days of incubation in all tested isolates with the initial DCF concentration (Table 1). The highest calculated degradation percentages were recorded for isolate S11, followed by isolate S18, while isolate S2 had the lowest degradation percentage of 12.85 among the isolates. A prolonged six-day incubation increased DCF degradation, and isolate S11 almost completely degraded DCF at 97.79 ± 0.84 percent. Bacterial isolate S2 had the minimum degradation percentage of 38.99 after six days of incubation. The bacteria-free control showed almost no change after three and six days of incubation under the same experimental conditions.

### 3.4. Identification of DCF-Degrading Bacterial Isolates

**Figure 2:**
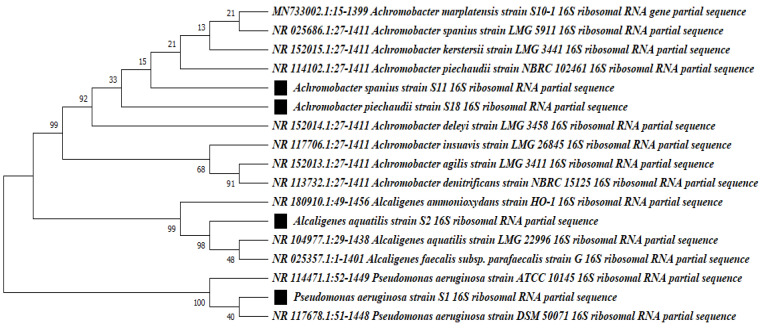
Maximum likelihood phylogenetic tree for 16S rRNA gene sequences of the best diclofenac-degrading isolates, *Pseudomonas aeruginosa* S1, *Alacaligense aquatilis* S2, and *Achromobacter spanius* strains S11 and S18, with related bacterial species in GenBank. The identified isolates are labeled with black boxes. The tree was generated using MEGAX software Version 10.1.8. The values at the nodes are the percentage values given by bootstrap sample analysis (1000×).

The DCF-degrading bacterial isolates were initially identified by morphological characters and different biochemical tests. Interestingly, all isolates were motile Gram-negative, had a short rod shape, and were catalase- and oxidase-positive. Isolate S1 was a lactose fermenter, while isolates S2, S11, and S18 were non-fermentative bacteria and were first identified to belong to the *Alcaligenaceae* family. All isolates were identified by sequencing the 16S rRNA gene to determine the genus and the species. S1 16S rDNA amplicons exhibited 100% identity with 16S rDNA from *P. aeruginosa* strains DSM 50071, ATCC 10145, and NBRC 12689 in the rRNA database of GenBank. The S2 isolate exhibited 98.65 and 98.64% identity with 16S rDNA from *Alcaligenes aquatilis* LMG 22996 and *Alcaligenes faecalis* subsp. *parafaecalis* strain G, respectively, and therefore, S2 was identified as *Alcaligenes aquatilis*. Isolates S11 and S18 were identified as an *A. spanius* strain and *A. piechaudii*, respectively. S11 and S18 were highly associated with *A. spanius* strain LMG 5911, showing 99.72 and 99.78% identity, respectively, followed by *A. kerstersii* LMG 3441, *A. deleyi* LMG 3458, and *A. piechaudii* NBRC 102461, which showed percent identity ranging from 99.57 to 99.71. The sequences for isolates S1, S2, S1, and S18 were deposited into the GenBank database under accession numbers OQ504372, OQ504374, OQ504475, and OQ504476, respectively. The sequence analysis utilizing the program MEGAX generated the phylogenetic tree with the related species, with 1000 bootstrap analyses, and revealed that the tree was divided into two main groups: the *P. aeruginosa* strain group and *Alacaligense group,* as well as an *Achromobacter* species group (Figure 2). The latter group was divided into two different subgroups, one for *Alacaligense* spp. and the other for *Achromobacter* spp.

### 3.5. Detection of DCF Biodegradation Intermediate Compounds Produced by Each Bacterial Strain

#### 3.5.1. Identification of Intermediate DCF Products of *A. spanius* S11 and *A. piechaudii* S18

**Figure 3:**
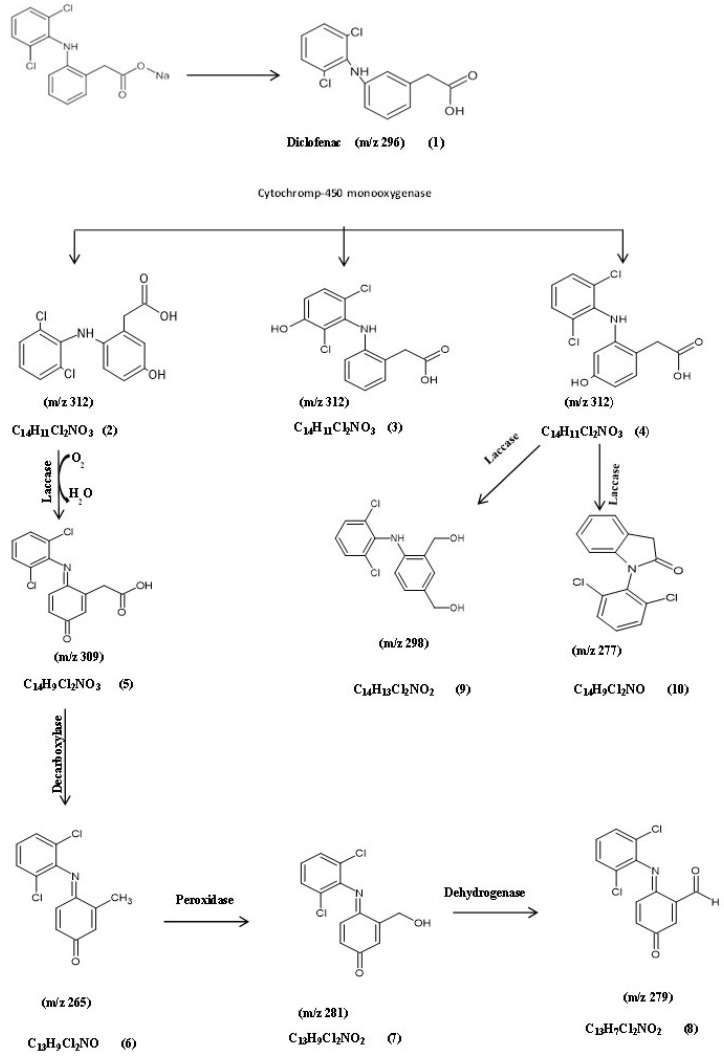
The postulated biodegradation pathway of diclofenac by *Achromobacter spanius* S11. (1) Diclofenac, (2) 5-hydroxydiclofenac, (3) 4-hydroxydiclofenac, (4) 3-hydroxy-diclofenac, (5) DF-2,5-benzoquinone imine, (6) (4E)-4-[(2,5-dichlorophenyl)imino]-3-methylcyclohexa-2,5-dien-1-one, (7) (4E)-4-[(2,5-dichlorophenyl)imino]-3-(hydroxymethyl)cyclohexa-2,5-dien-1-one, (8) (6E)-6-[(2,5-dichlorophenyl)imino]-3-oxocyclohexa-1,4-diene-1-carbaldehyde, (9) 4-(2,6-dichlorophenylamino)-1,3-benzenedimethanol, and (10) 1-(2,6-dichlorophenyl)indolin-2-one diclofenac amide.

Based on the previous biodegradation results, the most efficient isolates for degradation were *A. spanius* S11, *A. piechaudii* S18, and *P. aeruginosa* S1, so they were further characterized. To detect and identify biodegradation metabolites, the GC-MS technique was performed for each bacterial strain. The analysis of the GC-MS production profile for *A. spanius* S11 revealed that there were nine main metabolites detected among the experimental (actual) and predicted degradation metabolites as compared to the parent diclofenac, with a molecular weight (*m*/*z*) of 296 and a retention time (RT) of 12.77 min, which were the same as those of the authentic standard of diclofenac listed in the library databases and the control (pure) compound (Table 2, Table 3 and Table 4). In addition, a monohydroxylated product with a mass of *m*/*z* 312 was detected, which could be attributed to the addition of 16 mass units, revealing the formation of a singlet OH adduct. In general, the specificity of electrophilic aromatic substitution is typically driven by the nature of the substitute, which may account for our report of three different products with the same *m*/*z* ratio (312), confirming the molecular composition of C_14_H_11_C_l2_NO_3_ and corresponding to 5-hydroxy diclofenac (2), 4/-hydroxy diclofenac (3), and 3-hydroxy diclofenac (4), indicating the role of cytochrome-450 (CYP450) monooxygenase as the key enzyme in the biotransformation of diclofenac by *A. spanius* S11 (Figure 3). Subsequently, the loss of (-H_2_) from the molecular ion resulted in the formation of a fragment ion at *m*/*z* 309 with the molecular composition C_14_H_9_C_l2_NO_3_ (5), which is a quinone imine derivative of 5-hydroxy diclofenac (5-OH-DCF) generated by the action of laccase. The 5-OH-DCF quinone imine derivative (DF-2,5-benzoquinone imine) was the starting point for further multistep degradation involving decarboxylation, hydroxylation, and oxidation reactions. These mechanisms help to explain the presence of the secondary product metabolites at *m*/*z* 265 with the molecular formula C_13_H_9_C_l2_NO (6), which was converted to metabolite 7 with MW 281, or the molecular formula C_13_H_9_ C_l2_NO_2_ via the action of peroxidase and a fragment ion at *m*/*z* 279 with the molecular composition C_13_H_7_C_l2_NO_2_ (8). Another major metabolite was detected at *m*/*z* 298, which corresponds to the formation of metabolite 9 with the molecular formula C_14_H_13_C_l2_NO_2_ with spectral fragments of 4-(2,6-dichlorophenylamino)-1,3-benzenedimethanol. Finally, a fragment at *m*/*z* 277 corresponds to the formation of metabolite 10 with the molecular formula C_14_H_9_C_l2_ NO with spectral fragments of 1-(2,6-dichlorophenyl) indolin-2-one diclofenac amide (Table 2). Based on these results, a DCF catabolic degradation pathway was postulated (Figure 3).

**Figure 4:**
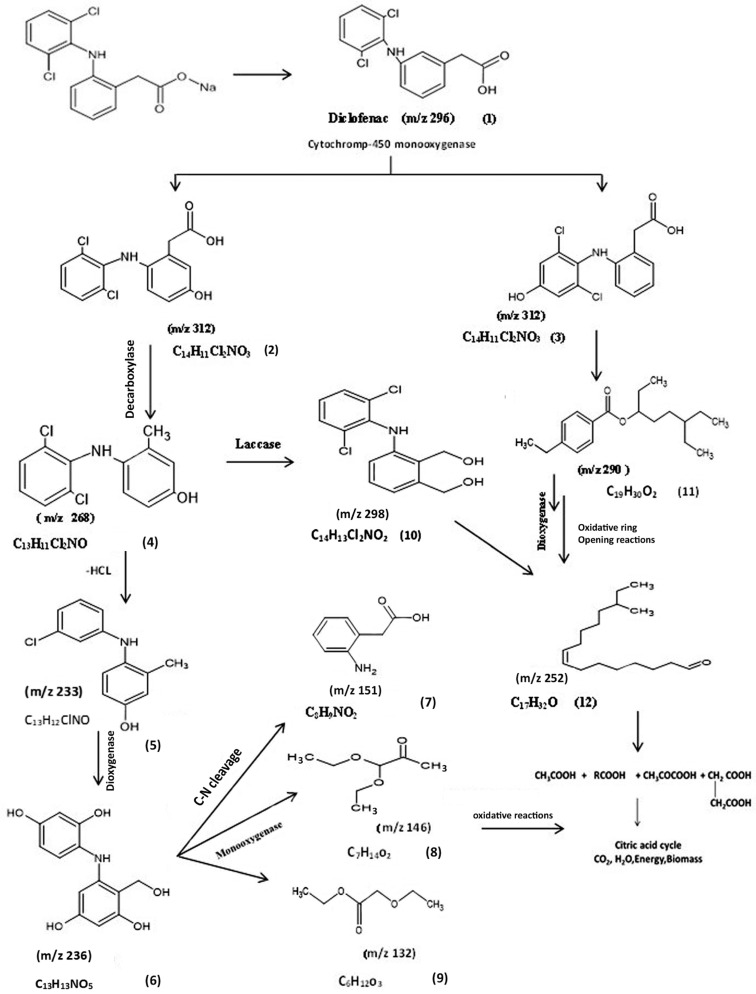
The proposed biodegradation pathway of diclofenac by *Achromobacter piechaudii* S18. (1) Diclofenac, (2) 5-hydroxydiclofenac, (3) 4/-hydroxydiclofenac, (4) (2, 4-dichloro-3-(2-methylanilino) phenol, (5) 3-(3-chloroanilino)-2-methylphenol, (6) 5-(2-dihydroxyanilino)-4-(hydroxymethyl) benzene-1,3-diol, (7) (2-aminophenyl)acetic acid, (8) pyruvaldehyde, 1 (diethyl acetal), (9) 2-ethoxyethyl acetate, (10) 4-dichlorophenylamino-1,3benzen dimethanol, (11) 4-ethylbenzoic acid, 6-ethyl-3-octyl ester, and (12) 4-methyl-8-hexadecenal.

On the other hand, the biodegradation of DCF with the *A. piechaudii* S18 isolate was different, suggesting divergent degradation pathways. Although the degradation rates were lower with *A. piechaudii* S18 than with *A. spanius* S11, it was more efficient in breaking down diclofenac into simpler compounds, which are summarized in Table 3. Figure 4 proposes the biodegradation pathway of *A. piechaudii* S18 based on the 11 main metabolites detected by the experimental (actual) and predicted degradation metabolites. Compared to the parent compound (diclofenac, *m*/*z* 296) (1), intermediate products with the same *m*/*z* ratio (312) at two different retention times (6.32 and 6.54) confirmed the molecular composition of C_14_H_11_Cl_2_NO_3_, corresponding to 5-hydroxydiclofenac (2) and 4-hydroxydiclofenac (3), indicating the presence of CYP450 monooxygenase as the key enzyme in DCF mineralization, and the subsequent hydroxydiclofenacan was dechlorinated through the loss of CO_2_, leading to the formation of the decarboxylated derivative of the monohydroxyl-DCF metabolite (3) at *m*/*z* 268 with the molecular formula C1_3_H_11_NCl_2_ (4), corresponding to (2,4-dichloro-3-(2-methylanilino) phenol.

The subsequent dichlorination (-HCl^-^) of the molecular ion resulted in the formation of a fragment ion at *m*/*z* 233 with the molecular formula C_13_H_12_ClNO, corresponding to the formation of 3-(3-chloroanilino)-2-methylphenol (metabolite 5), followed by the oxidation of the methyl group, hydroxylation, and the formation of metabolite 5 at *m*/*z* 263 with the molecular formula C_13_H_13_NO_5_, which corresponds to 5-(2,4-dihydroxyanilino)-4-(hydroxymethyl) benzene-1,3-diol. The fragment at *m*/*z* 151 corresponds to the cleavage of the NH bridge between the aromatic rings and the formation of metabolite 6 with the molecular formula C_8_H_9_NO_2_ with spectral fragments of (2-aminophenyl) acetic acid (metabolite 7). Metabolites 8 and 9 were the result of ring cleavage and the formation of a fragment at *m*/*z* 146 with the molecular formula C_7_H_14_O_3_ with spectral fragments typical of pyruvaldehyde, 1-(diethyl acetal), and a fragment at *m*/*z* 132 with the molecular formula C_7_H_14_O_3_ (metabolite 8) and 2-ethoxyethyl acetate (metabolite 9) with the molecular formula C_6_H_12_O_3_ (*m*/*z* 132.00).

A major metabolite occurred transiently and was identified by GC-MS as 4-dichlorophenylamino-1,3 benzendimethanol for a fragment ion at *m*/*z* 298 with the molecular formula C_14_H_13_Cl_2_NO_2_ (metabolite 10), indicating the role of the laccase enzyme in this transformation step. However, the presence of metabolite 11 with a fragment ion at *m*/*z* 290 and the molecular formula C_19_H_30_O_2_ and metabolite 12 with a fragment ion at *m*/*z* 252 and the molecular formula C_17_H_32_O, in addition to metabolites 8 and 9, clearly indicated the ability of *A. piechaudii* S18 to utilize DCF as the sole carbon source through a strong oxidative enzyme system (Figure 4). Therefore, the enzyme activities of both *Achromobacter* strains were further analyzed to support our suggested transformation map of diclofenac.

#### 3.5.2. Identification of Intermediate DCF Products of *P. aeruginosa* S1

**Figure 5:**
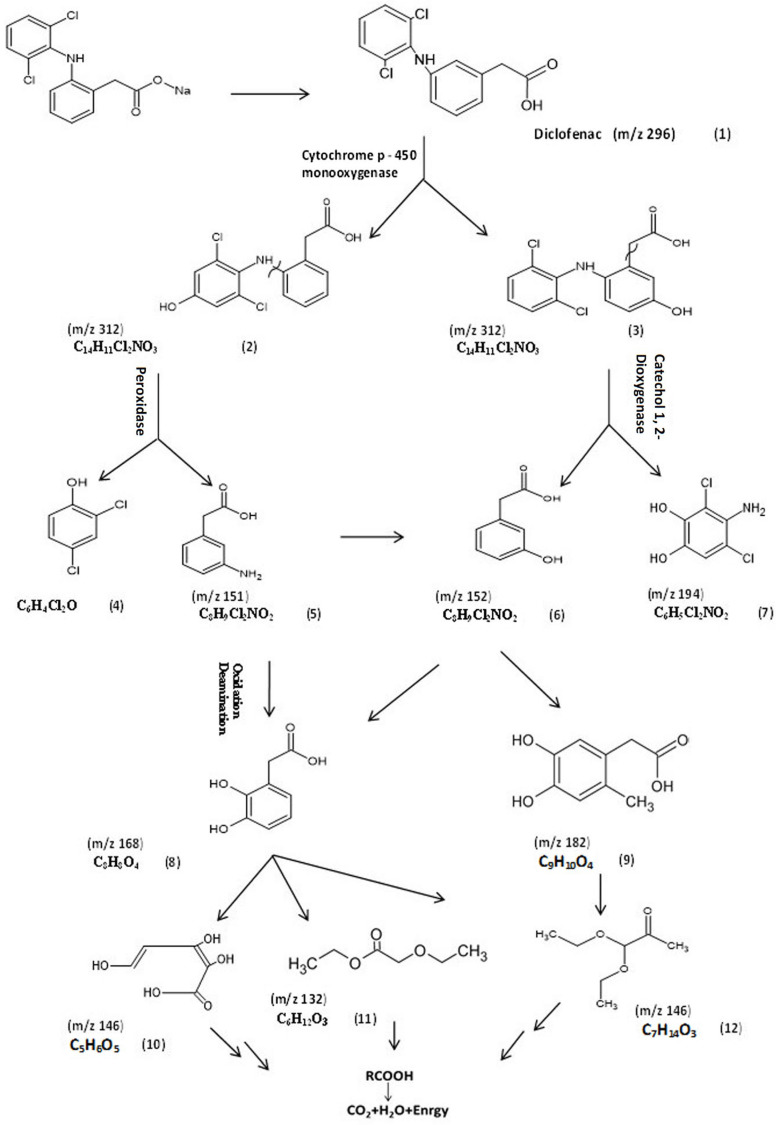
The proposed biodegradation pathway of diclofenac by *P. aeruginosa* S1. (1) Diclofenac, (2) 4/-hydroxydiclofenac, (3) 5-hydroxydiclofenac, (4) 2,4-dichlorophenol, (5) (3-aminophenyl)acetic acid, (6) (3-hydroxyphenyl)acetic acid, (7) 4-amino-3,5-dichlorobenzene-1,2-diol, (8) (2,3-dihydroxyphenyl)acetic acid, (9) (4,5-dihydroxy-2-methylphenyl)acetic acid, (10) (2Z,4E)-2,3,5-trihydroxypenta-2,4-dienoic acid, (11) ethyl ethoxyacetate, and (12) pyruvaldehyde, 1-(diethyl acetal).

To understand the mechanism of DCF by *P. aeruginosa* S1, the GC/MS chromatogram was analyzed and interpreted, as presented in Figure 5. The chemical structures of these products were elucidated from the mass library, as well as from their mass fragmentation patterns (Table 4). The expected initial step for the biodegradation of diclofenac was hydroxylation, resulting in 4/-hydroxydiclofenac (2) and 5-hydroxydiclofenac (3), indicating that CYP450 monooxygenase played the key role in the first step of DCF degradation by *P. aeruginosa* S1. As a consequence of hydroxylation, the second step was C-N cleavage between the aromatic rings, followed by a hydroxylation reaction: the key metabolites formed may be 2,4-dichlorophenol (4), as an expected compound for this step; (3-aminophenyl) acetic acid (5); (3-hydroxyphenyl) acetic acid (6); and 4-amino-3,5-dichlorobenzene (7). Another two major metabolites were detected at *m*/*z* 168, corresponding to (2,3-dihydroxyphenyl) acetic acid (8), and *m*/*z* 182 with the molecular formula C_9_H_10_O_4_, corresponding to (4,5-dihydroxy-2-methylphenyl) acetic acid (9), which verifies the assumption of hydroxyl substitution. Consequently, the results show another intermediate metabolite at *m*/*z* 146 with the molecular formula C_5_H_6_O_5_ that corresponds to (2Z,4E)-2,3,5-trihydroxypenta-2,4-dienoic acid (10); a metabolite at *m*/*z* 132 with the molecular formula C_9_H_10_O_4_, corresponding to ethyl ethoxyacetate (11); and a metabolite at *m*/*z* 146 with the molecular formula C_7_H_14_O_3_, corresponding to pyruvaldehyde, 1-(diethyl acetal) (12). Then, the further degradation of ethyl ethoxyacetate and pyruvaldehyde, 1-(diethyl acetal) produced CO_2_ and water. Therefore, DCF biodegradation by *P. aeruginosa* S1 was catalyzed by three major enzymes: cytochrome-450 monooxygenase, peroxidase, and catechol 1,2-dioxygenase. Accordingly, the enzyme activities of the three bacterial isolates were further analyzed to support our suggested diclofenac transformation map of diclofenac.

### 3.6. Specific Activities of Enzymes Involved in DCF Degradation

To reveal DCF microbial degradation enzymes, the specific activities of selected enzymes were determined for *A. spanius* S11, *A. piechaudii* S18, and *P. aeruginosa* S1. Hence, the specific activities of the selected enzymes (catechol 1,2-dioxygenase, catechol 2,3-dioxygenase, laccase, and peroxidase) were determined both in the presence of DCF and in the control medium (glucose instead of DCF). As shown in Table 5, *A. piechaudii* S18 was superior in catechol 1,2-dioxygenase activity (2.714 ± 0.147 U/mg), followed by *P. aeruginosa* S1 (1.832 ± 0.20 U/mg protein) and *A. spanius* S11 (0.928 ± 0.026 U/mg).

Furthermore, the catechol 2,3-dioxygenase assay demonstrated that the highest enzyme-specific activity was achieved with *P. aeruginosa* S1 in the presence of DCF (0.386 ± 0.032 U/mg), followed by *A. piechaudii* S18, with a specific activity of 0.334 ± 0.097 U/mg, unexpectedly less than in the control medium (with glucose). The lowest catechol 2,3-dioxygenase activity was detected with *A. spanius* S11. The opposite trend was obtained with laccase activity, where the highest activity was observed with *A. spanius* S11 and *A. piechaudii* S18 in the presence of DCF (0.807 ± 0.127 and 0.672 ± 0.096 U/mg protein, respectively), while no activity was detected for *P. aeruginosa* S1. Regarding peroxidase activity, the highest activity was observed with *P. aeruginosa* S1 in the presence of DCF (0. 880 ± 0.127 U/mg), followed by *A. piechaudii* S18 (0.747 ± 0.054 U/mg), while the lowest specific peroxidase activity was achieved with *A. spanius* S11 (0.107 ± 0.076 U/mg).

## 4. Discussion

The global contamination of different water resources with different pharmaceutical compounds is alarming due to their serious and chronic biological effects, such as lipophilicity and toxic effects that may occur in living beings [9,24]. DCF is one of the major pharmaceutical compounds detected in aquatic environments in different countries due to its high persistence and relatively high concentrations reported in surface and groundwater, as well as effluent water discharged from WWTPs [9,10]. The natural biodegradation of DCF in the ecosystem is mainly accomplished by bacteria and other microorganisms. Therefore, the bacteria-based biodegradation of pharmaceutical compounds is a promising area of research and a cost-effective strategy, especially for micropollutants. In this study, four bacterial isolates (S1, S2, S11, and S18) were able to grow in the presence of DCF as a sole source of carbon and energy (without a co-substrate), which provides evidence that these strains can use DCF as the sole carbon source, resulting in its biodegradation. The selected bacterial strains could tolerate exposure to high concentrations of DCF, up to 40 mg/L, at different magnitudes. It was observed that the selected bacterial strains required a prolonged lag phase when increasing the DCF concentration, and then the growth rate became stationary after six days of incubation. This suggests that DCF induces cellular toxicity, and each bacterial isolate has a different level of resistance. The high concentration of DCF induces the exacerbation of an oxidative burst inside the bacterial cells due to the liberation of reactive oxygen species (ROS) [12,25]. In this regard, ROS intermediate generation after the treatment of cancer cell lines was attributed to the inhibition of the cellular antioxidant enzyme superoxide dismutase [26]. Moreover, ROS can also trigger the bacterial membrane’s unsaturated fatty acids, as well as cellular proteins and DNA. A high concentration of the lipid peroxidation biomarker (MDA) was formed during bacterial exposure to DCF [7]. Bacteria can scavenge increased ROS inside the cells by using different antioxidant enzymes. This response to DCF exposure is specific to each bacterial strain [25]. Interestingly, the tested DCF concentration is higher than most of those reported in the previous literature for DCF degradation by a single bacterial strain, indicating the high efficiency of the scavenging systems of the selected isolates to cope with the oxidative stress induced by DCF [7,13,14]. However, a microbial consortium from forest soil samples completely degraded an elevated DCF concentration (100 mg/L) after only 10 days of aerobic incubation [27]. Remarkably, *Klebsiella* sp. KSC had the best degradation rate after only 72 h with an initial concentration of 70 mg/L [28]. The efficiency of the selected isolates was confirmed by the disappearance of the parent compound, with the maximum degradation percentage reaching 97.79 ± 0.84 for isolate S11 after only six days of incubation. These results are a significant improvement compared to 25 days for *Labrys portucalensis* F11 [14] and 12 days for the *Pseudomonas moorei* KB4 strain [25] for the complete degradation of a comparable concentration of DCF. Fast degradation rates were reported for *Rhodococcus ruber* strain IEGM 346 in six days [20] and *Bacillus subtilis* and *Brevibacillus laterosporus* strains after 17 h [29] but with a low initial concentration (50 µg/L) or with the intermediate product 4′-hydroxy-diclofenac, respectively.

The selected strains were capable of growing in a temperature range of 28–37 °C. This is in line with the reported literature for mesophilic bacteria that have an optimal growth temperature range of 35 to 37 °C [10].

pH optimization during exposure to DCF may have an additional advantage for the degradation process. It was observed that the optimal pH values for the growth of DCF-degrading bacteria were different among bacterial strains. Three isolates preferred pH values that were more alkaline (7–8), and only isolate S2 grew better at pH 5. Indeed, it was reported that for some bacterial strains, the intermediate metabolites of DCF degradation produced different types of amines that increased the pH value of the culture [7].

The identification of the selected isolates demonstrated that all isolates were Gram-negative. Three bacterial isolates belonged to the *Alcaligenaceae* family, and one bacterial isolate belonged to the *Pseudomonadaceae* family. In the literature, different genera of both Gram-positive and Gram-negative bacteria were identified as potential DCF-degrading bacteria, such as *Klebsiella*, *Raoultella*, *Bacillus*, and *Rhodococcus* [20,28,30]. Despite the diversity of the identified bacterial strains, few bacterial isolates were able to completely degrade DCF due to the difficulty of degradable intermediates generated during biodegradation [31]. In this study, two strains of the four selected bacteria were new for DCF biodegradation, namely, *A. spanius* strain S11 and *A. piechaudii* S18. Recently, some studies demonstrated the potentiality of *Achromobacter* sp. for the degradation of different types of organic pollutants, such as sulfamethoxazole by *Achromobacter* sp. JL9 [32] and petroleum bioremediation by *Achromobacter* sp. HZ01 [33]. Therefore, their degradation pathways were investigated alongside the well-known *P. aeruginosa* S1.

The results obtained from the identification of intermediate metabolites by GC-MS during DCF biodegradation suggested that the bacterial degradation reactions that occurred were mostly multidirectional [31]. The primary hydroxylation of DCF is considered a bottleneck step in DCF metabolites, and the detection of 4′-OH-DCF and 5′-OH-DCF in all investigated isolates indicated that CYP450 monooxygenase is a key enzyme in this degradation pathway [14,30]. In fact, most of the described microorganisms capable of DCF biodegradation transformed it into a more toxic hydroxylated form. However, only a few isolates could further cleave the aromatic structure of this hydroxylated DCF [2,31]. The formation of quinone imine derivatives of 5-hydroxy diclofenac (DF-2,5-QI) in the *A. spanius* S11 culture via the action of laccase was previously demonstrated [34]. This was confirmed by the high laccase activity observed in cells grown with DCF as a sole carbon source compared to control cells grown in glucose. However, the two-step biotransformation to DF-2,5-QI during the human metabolism of DCF demonstrated catalysis by two different CYPs: the highest activity was for the 5-hydroxylation of DF, which was predominantly catalyzed by CYP3A4, while its subsequent bioactivation to DF-2,5-QI was catalyzed by CYP2C9 [35]. The further degradation of DF-2,5-QI by *A. spanius* S11 may proceed via different pathways (decarboxylation, hydroxylation, and oxidation reactions). The DCF degradation described for *Labrys portucalensis* F11 proceeded mainly by hydroxylation reactions, where the formation of benzoquinoneimine species seems to be a central step in the degradation pathway [14]. The formation of 4-(2,6-dichlorophenylamino)-1,3-benzenedimethanol (metabolite 8) at *m*/*z* 298 via the action of the laccase enzyme was previously confirmed by the action of purified fungal laccase from the white rot fungus *Trametes versicolor* during DCF degradation [36]. The subsequent formation of 1-(2,6-dichlorophenyl) indolin-2-one diclofenac amide (metabolite 9) by *A. spanius* S11 could be attributed to the action of the laccase enzyme. The degradation of DCF with immobilized laccase on graphene oxide led to the identification of the same compound as a major metabolite in the degradation of DCF [37]. On the other hand, the complete degradation and disturbance of the aromatic structure of hydroxydiclofenac was observed with the isolated strain *A. piechaudii* S18. This compound was dechlorinated through CO_2_ loss, which formed 5-(2,4-dihydroxyanilino)-4-(hydroxymethyl) benzene-1,3-diol (metabolite 4), where dichlorination is the central step for degradation and decreases the ecotoxicity of DCF metabolites [38,39]. Ring cleavage occurred subsequent to metabolite 5, producing pyruvaldehyde, 1-(diethyl acetal) and 2-ethoxyethyl acetate due to subsequent oxidation followed by the cleavage of the N-C bond and the formation of 5-(2,4-dihydroxyanilino)-4-(hydroxymethyl) benzene-1,3-diol (metabolite 6). The detection of 4-dichlorophenylamino-1,3 benzen dimethanol (metabolite 10) produced by the action of laccase was previously reported in a study performed on DCF using commercial laccase from *Trametes versicolor* [2]. Many studies correlated the laccase activity with the microbial degradation of DCF and suggested that the presence of nitrogen and the negative charge in the DCF ring play important roles in the degradation of these compounds by the laccase enzyme [40,41]. Furthermore, the formation of by-products without an aromatic structure (metabolites 8, 9, and 12) allows their further incorporation into the Krebs cycle [25]. Taken together, these results indicate that most of the detected metabolites generated by this bacterial strain are less toxic than the parent compound DCF.

The DCF biodegradation pathway of *P. aeruginosa* S1 was mostly multidirectional, as confirmed by the detection of 11 main metabolites [42]. The C-N cleavage between aromatic rings, followed by a hydroxylation reaction, led to the formation of key metabolites, including 2,4-dichlorophenol (4), (3-aminophenyl) acetic acid (5), (3-hydroxyphenyl) acetic acid (6), and 4-amino-3,5-dichlorobenzene (7). The hydroxyl derivative was exposed, leading to the subtraction of an electron and H^+^ from the hydroxyl phenolic group by peroxidases, generating a phenoxyl radical [43]. Peroxidase could catalyze the first oxidative dechlorination step in the degradation of several chlorinated phenols. When the electrophilic hydroxyl radical was added to the aromatic ring, a resonance-stabilized carbon-centered radical was formed, and the hydrogen radical was eliminated. With further hydroxyl radical oxidation, multi-dihydroxylation products were formed, including (3,5-dihydroxyphenyl) acetic acid (8) and (4,5-dihydroxy-2-methyl phenyl) acetic acid (9), which verify the assumption of hydroxyl substitution, indicating the role of catechol 1,2-dioxygenase, which was indirectly proven by the high enzymatic activity observed in *P. aeruginosa* S1. The hydroxyl group enhances the aromatic ring’s electron density; hence, hydroxyl radical electrophilic adductions occur more quickly [44]. Consequently, another intermediate metabolite at *m*/*z* 132 with the molecular formula C_9_H_10_O_4_, corresponding to ethyl ethoxyacetate (11), and a metabolite at *m*/*z* 146 with the molecular formula C_7_H_14_O_3_, corresponding to pyruvaldehyde, 1-(diethyl acetal) (12), were generated. Then, the further degradation of ethyl ethoxyacetate and pyruvaldehyde, 1-(diethyl acetal) produced CO_2_ and water. Indeed, recent work demonstrated that *P. aeruginosa* is capable of mineralizing many persistent aromatic pollutants, including chlorinated phenols [45,46]. However, DCF degradation was previously confirmed for *Pseudomonas moorei* but not for *P. aeruginosa* [25].

From the proposed degradation map, it can be concluded that the first steps of the oxygenation of DCF occur via phenol hydroxylase enzymes to form monohydroxyl-DCF, followed by peroxidase, which could catalyze the first oxidative dechlorination step and the subsequent ring cleavage adjacent to or in between the two hydroxyl groups of polyhydroxylated derivatives. Phenol hydroxylases ranging from simple flavoprotein monooxygenases to multicomponent hydroxylases, as well as the genes coding for these enzymes, were described for several aerobic phenol-degrading microorganisms [47].

The postulated degradation pathway of DCF by the three bacterial strains established the activities of monooxygenase, dioxygenase, peroxidase, and laccase enzymes generated during degradation by all bacterial species. Although many studies have reported DCF degradation, reports on the specific enzymatic activities of DCF-degrading bacteria are still scarce. Therefore, we investigated the enzymatic activities to integrate the biodegradation pathway with specific enzymatic activities. Catechol 1,2-dioxygenase, peroxidase, and laccase enzymes were induced in all isolates tested in the presence of DCF compared to control cells, confirming the involvement of these enzymes in the degradation process. However, catechol 2,3-dioxygenase activity was high in the glucose-free control culture of *A. piechaudii* S18. In this regard, the expression level of catechol 1,2-dioxygenases increased significantly (145 times) in the *Pseudomonas moorei* KB4 strain after DCF exposure, but up-regulation was not detected for catechol 2,3-dioxygenase [25].

The enhanced degradation activities of *A. spanius* S11 and *A. piechaudii* S18 are in line with the ability of different *Achromobacter* strains to produce monooxygenase and dioxygenase enzymes, such as *Achromobacter* sp. HZ01, *Achromobacter* sp. DMS1, and *Achromobacter xylosoxidans* DN002 [48]. The presence of several genes encoding the monooxygenase family, as well as the blue multicopper oxidase family, in the genome of *Achromobacter* sp. HZ01 strain confirmed the bioremediation potential of Achromobacter strains [33].

## Figures and Tables

**Table 1:** The percentages of diclofenac degradation in MS after incubation at 37 °C for three and six days.

Isolates	Degradation of Diclofenac (%) *	
	3 Days	6 Days
S1	37.76 ± 0.93 ^c^	75.20 ± 0.71 ^c^
S2	12.85 ± 1.58 ^b^	38.99 ± 0.90 ^b^
S11	86.60 ± 1.19 ^e^	97.79 ± 0.84 ^e^
S18	53.15 ±1.55 ^d^	88.72 ± 0.89 ^d^
Control	2. 29 ± 1.32 ^a^	3.51 ± 0.87 ^a^

* The initial diclofenac concentration in MS medium was 40 mg/L. The degradation percentage values are means of three independent replicates ± standard deviations. Means with different lower-case letters differ significantly at the probability level of 0.05, as analyzed by Tukey’s test.

**Table 2:** DCF and identified DCF transformation products detected by GC/MS during biodegradation by *Achromobacter spanius* S11.

No.	Compound Name	RT (min)	Fragments (*m*/*z*)	MW	Formula
1	Diclofenac	12.77	249, 277, 295	296	C_14_H_11_C_l2_N_2_O_2_
2	5-Hydroxydiclofenac	10.93	214, 231, 249, 312	312	C_14_H_11_Cl_2_NO_3_
3	4/-Hydroxydiclofenac				
4	3-Hydroxydiclofenac				
5	DF-2,5-benzoquinone imine	13.72	242, 279, 309	309	C_14_H_9_Cl_2_NO_3_
6	(6E)-6-[(2,5-dichlorophenyl) imino]-3-oxocyclohexa-1,4-diene-1-carbaldehyde	12.88	111, 222, 265	265	C_13_H_9_Cl_2_NO
7	(4E)-4-[(2,5-dichlorophenyl) imino]-3-(hydroxymethyl) cyclohexa-2,5-dien-1-one	--------	-----	281	C_13_H_9_Cl_2_NO_2_
8	(6E)-6-[(2,5-dichlorophenyl) amino]-3-oxocyclohexa-1,4-diene-1-carbaldehyde	7.66	242, 277, 278	279	C_13_H_7_Cl_2_NO_2_
9	4-(2,6-dichlorophenylamino)-1,3-benzenedimethanol	13.07	179, 214, 242, 279	298	C_14_H_13_Cl_2_NO_2_
10	1-(2,6-Dichlorophenyl)indolin-2-one diclofenac amide	13.72	151, 214, 242, 277	277	C_14_H_9_Cl_2_NO

**Table 3:** DCF and identified DCF transformation products detected by GC/MS during biodegradation by *Achromobacter piechaudii* S18.

No.	Compound Name	RT (min)	Fragments (*m*/*z*)	MW	Formula
1	Diclofenac	12.77	249, 277, 295	296	C_14_H_11_Cl_2_N_2_O_2_
2	5-Hydroxydiclofenac	6.32	214, 231, 249, 312	312	C_14_H_11_Cl_2_NO_3_
3	4^/^-Hydroxydiclofenac	6.54	214, 231, 249, 312	312	C_14_H_11_Cl_2_NO_3_
4	(2, 4-Dichloro-3-(2-methylanilino) phenol	7.15	249, 268, 176	268	C_13_H_11_Cl_2_NO
5	3-(3-Chloroanilino)-2-methylphenol	15.73	133, 203, 233, 107	233	C_13_H_12_ClNO
6	5-(2,4-Dihydroxyanilino)-4-(hydroxymethyl) benzene-1,3-diol	4.6	133, 151, 203, 263	263	C_13_H_13_No_5_
7	(2-Aminophenyl) acetic acid	5.04	77, 133, 151	151	C_8_H_9_NO_2_
8	Pyruvaldehyde, 1-(diethyl acetal)		47, 101, 144, 146	146	C_7_H_14_o_2_
9	2-Ethoxyethyl acetate	4.6	59, 88, 103, 132	132	C_6_H_12_o_3_
10	4-Dichlorophenylamino-1,3benzen dimethanol	14.67	214, 242, 277, 298	298	C_14_H_13_Cl_2_NO_2_
11	4-Ethylbenzoic acid, 6-ethyl-3-octyl ester		55, 70, 135, 290	290	C_19_H_30_O_2_
12	4-Methyl-8-hexadecenal, Z	7.17	55, 70, 135, 252	252	C_17_H_32_O

**Table 4:** DCF and identified DCF transformation products after degradation by *P. aeruginosa* S1.

No.	Compound Name	RT (min)	Fragments (*m*/*z*)	MW	Formula
1	Diclofenac	33.66	249, 277, 295	296	C_14_H_11_Cl_2_N_2_O_2_
2	4^/^-Hydroxydiclofenac	6.26	214, 231, 249, 312	312	C_14_H_11_Cl_2_NO_3_
3	5-Hydroxydiclofenac	6.67	214, 231, 249, 312	312	C_14_H_11_Cl_2_NO_3_
4	2,4-Dichlorophenol	-------	-------	163	C_6_H_4_Cl_2_O
5	(3-Aminophenyl) acetic acid	5	51, 77, 133, 151	151	C_8_H_9_NO_2_
6	(3-Hydroxyphenyl) acetic acid	5.21	77, 133, 151	152	C_8_H_8_O_3_
7	4-Amino-3,5-dichlorobenzene	5	151, 179, 194	194	C_6_H_5_Cl_2_NO_2_
	-1,2-diol				
8	(2,3-Dihydroxyphenyl) acetic acid	7.19	55, 124, 168	168	C_8_H_8_O_4_
9	(4,5-Dihydroxy-2-methylphenyl) acetic acid	7.19	151, 179, 182	182	C_9_H_10_O_4_
10	(2Z,4E)-2,3,5-Trihydroxypenta-2,4-dienoic acid	9.18	47, 101, 144	146	C_5_H_6_O_5_
11	Ethyl ethoxyacetate	4.57	31, 59, 88, 103, 132	132	C_6_H_12_O_3_
12	Pyruvaldehyde,1-(diethyl acetal)	9.14	47, 101, 144	146	C_7_H_14_O_3_

**Table 5:** Specific activity of selected enzymes (U/mg protein) measured for *Achromobacter spanius* S11, *Achromobacter piechaudii* S18, and *P. aeruginosa* (S1) in the presence of DCF or glucose as a control.

Enzyme	Specific Enzyme Activity (mU/mg Protein)					
	*A. spanius* S11		*A. piechaudii* S18		*P. aeruginosa* S1	
	Control *	MS + DCF **	Control	MS + DCF	Control	MS + DCF
Catechol 1,2-dioxygenase	0.777 ± 0.064	0.928 ± 0.026	1.34 ± 0.256	2.714 ± 0.147	1.137 ± 0.293	1.832 ± 0.20
Catechol 2,3-dioxygenase	0.0021 ± 0.008	0.005 ± 0.002	0.334 ± 0.097	0.131 ± 0.083	0.059 ± 0.016	0. 386 ± 0.032
Laccase	0.192 ± 0.037	0.807 ± 0.127	0.390 ± 0.036	0.672 ± 0.096	0.004 ± 0.002	0.002 ± 0.001
Peroxidase	0.0457 ± 0.04	0.107 ± 0.076	0.654 ± 0.041	0.747 ± 0.054	0. 328 ± 0.071	0. 880 ± 0.127

* Control is minimal salt medium supplemented with 10 mg/L glucose. ** MS is a minimal salt medium supplemented with 10 mg/L diclofenac as the sole carbon source.

## Data Availability

All data are included in the manuscript and the supplementary files.

## Supplementary Materials

The following supporting information can be downloaded at: https://www.mdpi.com/article/10.3390/microorganisms11061445/s1.
