# Supplementary material for: Complete Biodegradation of Diclofenac by New Bacterial Strains: Postulated Pathways and Degrading Enzymes
Source: Microorganisms. 2023 May 30;11(6):1445. doi: 10.3390/microorganisms11061445 (PMC10301663; doi:10.3390/microorganisms11061445)
Supplement: Supplementary file 1 [file microorganisms-11-01445-s001.zip › microorganisms-2419831-supplementary.pdf]

Supplementary Figures

Figure S1

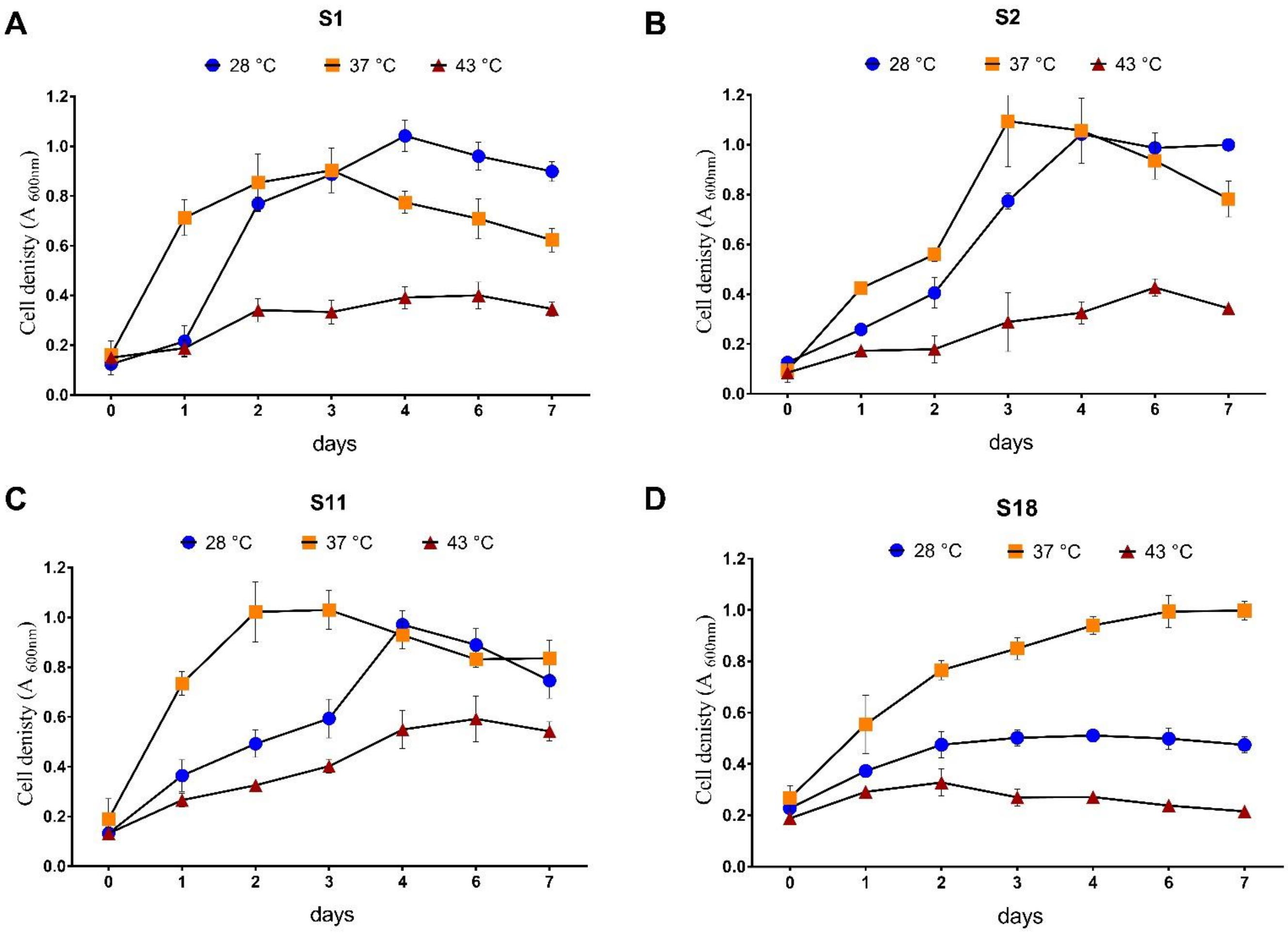

Figure S1. The effect of temperature on the growth of the four selected bacterial isolates (A; S1, B; S2, C; S11 and D; S18) determined at OD 600 nm after different time intervals (1-7 days) in minimal salt media supplemented with 30 mg/l as the sole carbon source. The curve represented by mean ± SD of three in dependent replicates.

Figure S2

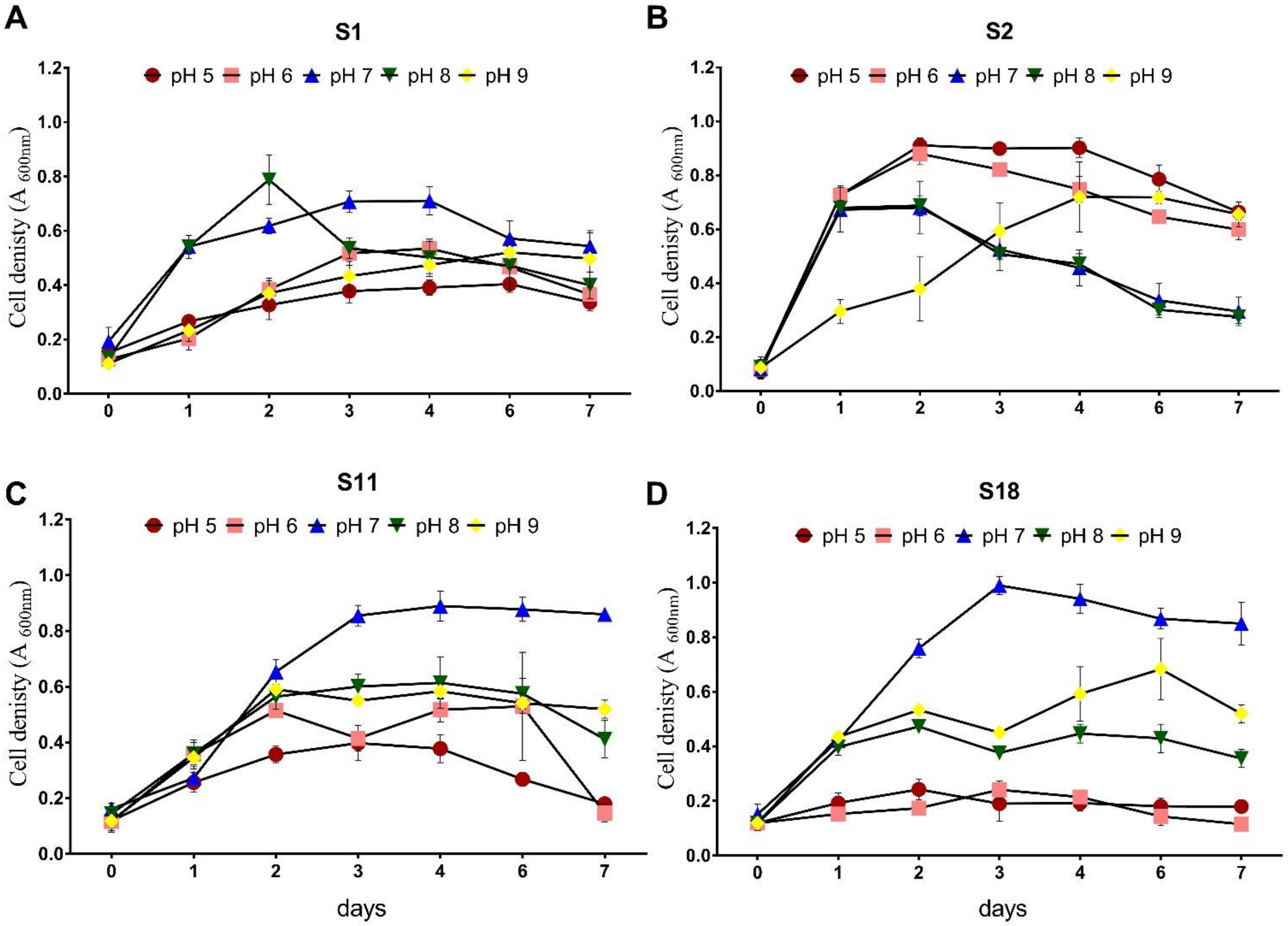

Figure S2. The effect of pH on the growth of the four selected bacterial isolates (A; S1, B; S2, C; S11 and D; S18) determined at OD 600 nm after different time intervals (1-7 days) with an initial diclofenac concentration of 30 mg/l as the sole carbon source in minimal salt media. The curve represented by mean ± SD of three in dependent replicates.

Figure S3

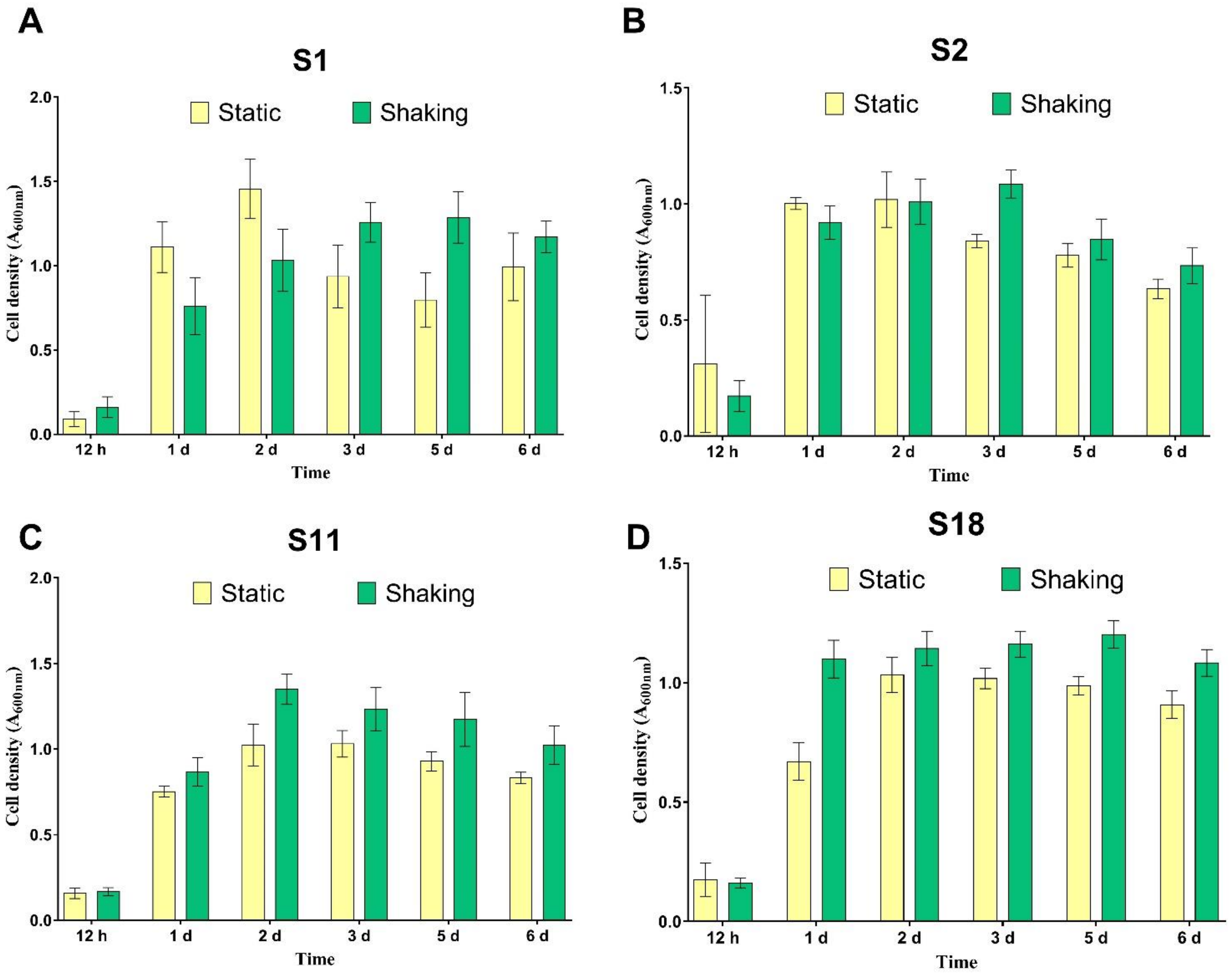

Figure S3. The effect of static and shaking incubation experiments on the performance of selected bacterial isolates (A; S1, B; S2, C; S11 and D; S18) determined at OD 600 nm after a different time intervals (1-6 days) with initial diclofenac concentration 30 mg/l as the sole carbon source in minimal salt medium. The bars are represented by mean ± SD of three independent replicates.

Figure S4

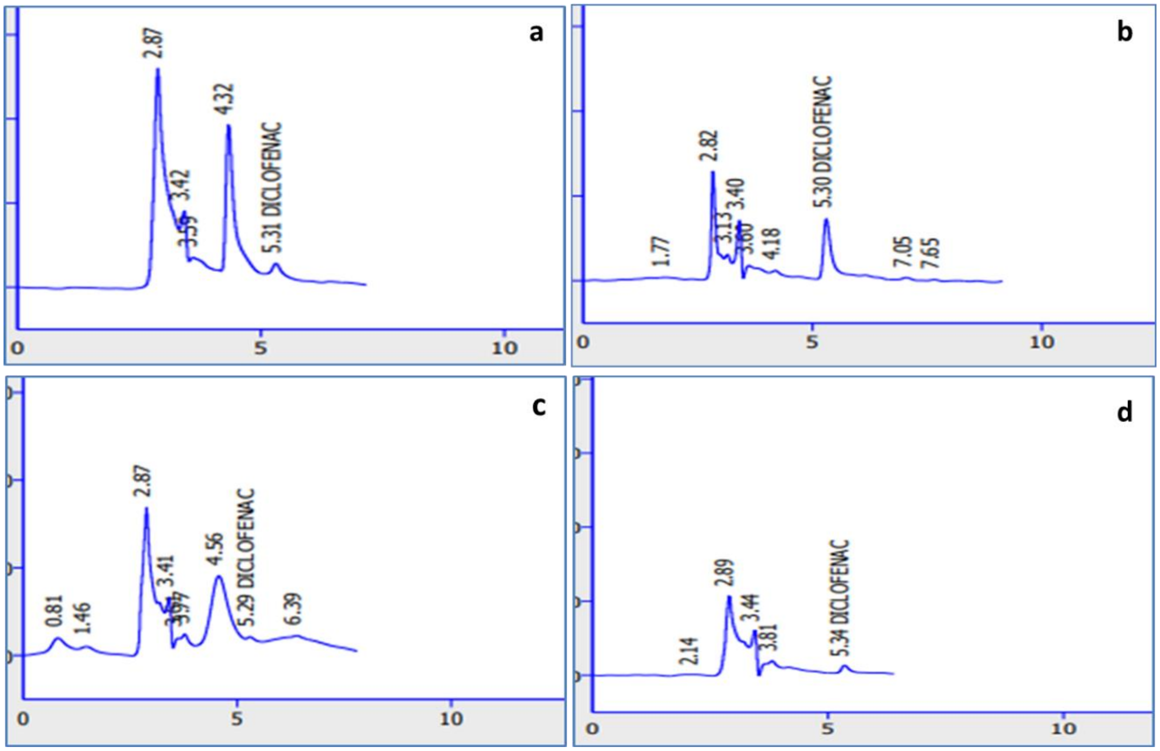

Figure S4. The HPLC chromatogram for the biodegradation of diclofenac in minimal salt medium with initial concentration of diclofenac 30 mg/L by (a) *P. aeruginosa* S1 (b) *Alacaligense aquatilis* S2 (c) *Achromobacter spanius* S11 and (d*) Achromobacter piechaudii* S18 after 6 days of incubation period.
